# The Protection of Midazolam Against Immune Mediated Liver Injury Induced by Lipopolysaccharide and Galactosamine in Mice

**DOI:** 10.3389/fphar.2018.01528

**Published:** 2019-01-08

**Authors:** Jian Li, Hong Tan, Xiaona Zhou, Chunpan Zhang, Hua Jin, Yue Tian, Xinyan Zhao, Xinmin Li, Xuelian Sun, Meili Duan, Dong Zhang

**Affiliations:** ^1^Department of Intensive Care Unit, Beijing Friendship Hospital, Capital Medical University, Beijing, China; ^2^Beijing Clinical Research Institute, Beijing, China; ^3^Beijing Key Laboratory of Tolerance Induction and Organ Protection in Transplantation, Beijing, China; ^4^Department of Anesthesiology, Beijing Friendship Hospital, Capital Medical University, Beijing, China; ^5^General Surgery Department, Beijing Friendship Hospital, Capital Medical University, Beijing, China; ^6^Experimental and Translational Research Center, Beijing Friendship Hospital, Capital Medical University, Beijing, China; ^7^Department of Emergency Medicine, Beijing Friendship Hospital, Capital Medical University, Beijing, China; ^8^National Clinical Research Center for Digestive Diseases, Beijing, China

**Keywords:** immunity, inflammation, lipopolysaccharide, liver injury, macrophage, midazolam

## Abstract

**Objectives:** Liver macrophages agitated by Lipopolysaccharide (LPS) can enhance immuno-inflammatory responses in the liver which mediate liver injury and result in dysfunction. Midazolam has been reported to have inhibitory effects on activated immunity and escalated inflammation, however, what the effects of midazolam on the liver injury caused by excessive immuno-inflammatory response in sepsis, and what influence it will exert on inflamed liver macrophages need to be elucidated.

**Methods:** In the present study, LPS and galactosamine-induced acute liver injury mice were used to observe the effect of midazolam *in vivo*. LPS-stimulated bone marrow cells were used to evaluate the influence of midazolam on monocytes *in vitro*.

**Results:** Midazolam prevented liver tissue injury and decreased serum alanine transaminase (ALT) level in LPS plus galactosamine treated mice. Mechanistically, midazolam suppressed tumor necrosis factor-α (TNF-α) and interleukin-1β (IL-1β) produced by LPS stimulated liver macrophages *in vivo* and bone marrow monocytes *in vitro*, and reduced the expression of major histocompatibility complex class II (MHC II), cluster of differentiation 40 and 86 (CD40 and CD86) on the cell surface. These results could be reversed by PK-11195, a peripheral benzodiazepine receptor (PBR) blocker.

**Conclusion:** Midazolam can prevent liver from LPS-induced immune mediated liver injury by inhibiting inflammation and immune activation in liver macrophages.

## Introduction

Liver dysfunction is a common complication of sepsis with an approximate incidence of 40% (van Gestel et al., 2004; Cheng et al., 2007; Kobashi et al., 2013). The mortality of septic patients with liver dysfunction remains at 54%∼68%, which is higher than that of respiratory dysfunction in such patients (Cheng et al., 2007; Yan et al., 2014). In the process of sepsis, lipopolysaccharide (LPS) and the subsequently activated immunity and inflammation provoke liver macrophages in eliminating pathogens by phagocytosis, antigen presentation, high level cytokine and chemokine secretion, and oxygen and nitrogen radical production. However, these originally defensive reactions often result in liver tissue damage, which in turn can enhance the immuno-inflammatory responses in the injured liver. Thus, the reciprocal causation between the overreacted immuno-inflammatory responses and liver injury constructs a vicious circle and develops into liver dysfunction and even failure (Antoniades et al., 2008; Heymann and Tacke, 2016).

Midazolam, a benzodiazepine derivative, has been routinely used for sedation in critically ill patients in intensive care units. Recent studies have shown that midazolam depressed plasma levels of interleukin-1β, 6, 8 (IL-1β, 6, 8) and tumor necrosis factor-α (TNF-α) in critically ill patients (Helmy and Al-Attiyah, 2001). In murine models, midazolam reduced cluster differentiation 80 and 86 (CD80 and CD86), and major histocompatibility complex class II (MHC II) on dendritic cells and inhibited the proliferation of CD3+T cells and T helper 1 (Th1) cellular immune response (Ohta et al., 2011). Such evidence suggests that midazolam might be beneficial to septic patients who are suffering uncontrolled immuno-inflammatory responses. As for sepsis-induced liver injury, whether midazolam will aggravate liver dysfunction and what influence it might exert on liver macrophages during liver inflammation are still not clear. Therefore, we studied the effect of midazolam on acute liver injury induced by LPS and galactosamine in mice and investigated its effect on the immuno-inflammatory responses in liver macrophages *in vivo* and in bone marrow monocytes *in vitro*. Our results demonstrated that midazolam prevented acute liver injury and protected liver function mainly by inhibiting immuno-inflammatory responses in liver macrophages, especially in the liver infiltrating monocytes. A possible mechanism of midazolam could be, at least partially, due to blocking the nuclear factor-κB (NF-κB) signaling pathway after midazolam binding to the peripheral benzodiazepine receptor (PBR) in macrophages.

## Materials and Methods

### Mice

Male C57BL/6 wild type mice, which were 8-week-old and specific-pathogen-free, were purchased from Beijing Vital River Laboratory (Beijing, China). The mice were housed under pathogen-free conditions with a 12-h light-dark cycle and free access to food and water in the animal facilities at the Beijing Friendship Hospital. All experimental procedures were conducted in accordance with the protocol approved by the Institutional Animal Care and Use Committee, Beijing Friendship Hospital, Capital Medical University.

### Reagents and Antibodies

Midazolam injection (batch No. H19990027) was purchased from Jiangsu Nhwa Pharmaceutical Co., Ltd., (Jiangsu, China). *Escherichia coli* LPS (O111:B4) and galactosamine were purchased from Sigma-Aldrich (St Louis, MO, United States). PK11195 (ab109497) was purchased from Abcam (Cambridge, United Kingdom). Fluorochrome-conjugated antibodies against mouse CD45, CD11b, Ly6G, Ly6C, F4/80, TNF-a, CD86, MHC II, CD40, CC chemokine receptor 2 (CCR2), Biotin anti-IL-1β, streptavidin-APC, TER-119, GR1, and B220 were listed in the Supplementary Table S1.

### LPS and Galactosamine Induced Acute Liver Injury

Mouse acute liver injury was induced by intraperitoneal injection of LPS (5 ug/kg body weight) plus galactosamine (200 mg/kg body weight) (LG group, *n* = 5). To assess the effects of midazolam, mice were administered with midazolam intraperitoneally (8 mg/kg body weight) 30 min before and subcutaneously (4 mg/kg body weight) 30 min after the injection of LPS and galactosamine (MLG group, *n* = 5). Mice received only midazolam without LPS and galactosamine were regarded as the Mida group (*n* = 5). All reagents above were dissolved in normal saline (NS) at the indicated concentrations. NS was used as the blank vehicle in the mice of the Control group (*n* = 5). The mice were killed 12 h after the LPS and galactosamine injection. Blood and liver samples were harvested at the time of execution.

### Serum ALT Level

Serum ALT levels were measured using the Alanine Aminotransferase Assay Kit (Nanjing Jiancheng Bioengineering Institute, Jiangsu, China) according to the manufacturer’s instructions.

### Histological Analysis

Liver tissue was fixed in 4% paraformaldehyde overnight, embedded in paraffin and cut into 4-μm sections. The sections were stained with hematoxylin and eosin.

### Analysis of Immunocytes From Mouse Liver *in vivo*

The liver was perfused with NS by inserting a syringe into the left ventricle. Livers were excised, minced using a gentle MACS Dissociator (Miltenyi Biotec, Italy), and digested by collagenase IV (Sigma-Aldrich, United States)/DNase I (Roche, Germany), and then intrahepatic immunocytes were isolated and purified by percoll density gradient centrifugation according to standard procedures (Zhang et al., 2005). All cells were stained with the following fluorochrome-conjugated antibodies: phycoerythrin-Cyanine7 anti-CD45, fluorescein isothiocyanate anti-CD11b, allophycocyanin-Cy7 anti-Ly6G, allophycocyanin anti-F4/80. The experimental conditions of the antibodies are listed in Supplementary Table S1. Liver infiltrating monocytes (CD45^+^Ly6G^-^CD11b^high^F4/80^low^), Kupffer cells (CD45^+^Ly6G^-^CD11b^low^F4/80^high^) and liver neutrophils (CD45^+^CD11b^+^Ly6G^+^) subsets were examined on an Aria II flow cytometer with a fluorescence-activated cell sorter (FACS) (BD Biosciences, San Jose, CA, United States) from liver immunocytes. The gating strategy used for flow cytometry on liver immunocytes is shown in Supplementary Figure S1. The data were analyzed using FlowJo software (Treestar, Ashland, OR, United States).

### Bone Marrow (BM) Cell Culture and Treatment

BM cells were extracted from femurs and tibias of healthy C57BL/6 mice; red blood cells were removed by RBC lysis buffer (Qiagen, Valencia, CA, United States). Then, GR1+, TER119+, and B220+ cells were depleted using magnetically conjugated antibodies. After that, BM cells were suspended in complete medium (RPMI 1640 supplemented with 10% fetal bovine serum, 1% L-glutamine, 100U/ml penicillin, and 100 ug/ml streptomycin), and were seeded into 24-well-plates with the number of 3 × 10^6^/well. Cells were incubated in 95% air–5% CO_2_ humidified atmosphere at 37°C. In evaluation of the cell responses to midazolam and LPS, BM cells were cultured in complete medium with or without midazolam (100 uM) for total 16 h, and were challenged by LPS (100 ng/ml) in the last 4 of 16 h. BM cells incubated only in complete medium were regarded as the control. BM cells incubated in complete medium with midazolam only were regarded as the Mida group. In addition, to examine if cells could response to midazolam via the PBR, PK-11195 (100 uM), a selective antagonist of PBR, was applied 30 min before the incubation of midazolam followed by LPS stimulation. PK-11195 was first resolved in dimethyl sulfoxide (DMSO), and then diluted 1000-fold in complete medium to ensure the final concentration of DMSO would not exceed 0.2% (v/v) (Anderson et al., 2011).

### Real-Time Polymerase Chain Reaction (Real-Time PCR)

Total RNA was extracted from liver tissues and cultured cells using the RNeasy Mini-Kit (Qiagen, Valencia, CA, United States) and reverse transcribed to cDNA using the PrimeScript^TM^ RT Reagent Kit (TaKaRa Bio, Shiga, Japan). TNF-α, IL-1β, CCR2, inducible nitric oxide synthase (iNOS), and Arginase-1 (Arg-1), CD86, CD40, NF-κB (p105) and p65 (Rel A) mRNA were quantified by real-time PCR using the ABI 7500 Sequence Detection System (Applied Biosystems, Foster City, CA, United States). The expression of each gene was normalized to glyceraldehyde-3-phosphate dehydrogenase (GAPDH) and quantified using the 2^-ΔΔCt^ method. The primer sequences are shown in Supplementary Table S2.

### Analysis of BM Cells Cultured *in vitro*

The cultured BM cells were analyzed for the expression of various cell markers with flow cytometry analysis using intracellular staining. In brief, cells were fixed and permeabilized using intracellular fixation buffer and permeabilization buffer (eBioscience) according to the manufacturer’s instructions. All samples were run on an Aria II flow cytometer with a FACS (BD Biosciences) and the gating strategy used for flow cytometry on BM monocytes is shown in Supplementary Figure S2. The data were analyzed using FlowJo software (Treestar, Ashland, OR, United States).

### Statistical Analysis

The data were analyzed with GraphPad Prism 6.0 software (GraphPad Software, San Diego, CA, United States). The values were presented as the means ± standard deviation (*SD*). The difference among groups was examined by one-way ANOVA analysis and comparison between two groups was analyzed by *t*-test. *P*-values <0.05 were considered statistically significant.

## Results

### Midazolam Prevented Acute Liver Injury Induced by LPS and Galactosamine

**FIGURE 1:**
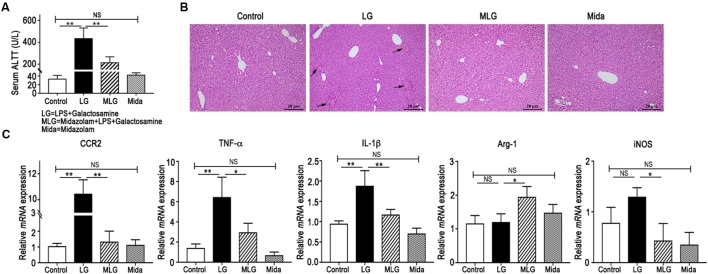
Midazolam prevented LPS and galactosamine induced acute liver injury in mice. **(A)** Serum ALT levels. **(B)** Hematoxylin and eosin stained liver tissue sections. The arrows indicate the spotty coagulation necrosis area in the liver of LG mouse. **(C)** Relative mRNA expressions in the liver tissues of mice in each group. The data are presented as the mean ± *SD*, *n* = 5 in each group. The difference among groups was examined by one-way ANOVA analysis and comparison between two groups was analyzed by *t*-test. ^∗^*p* < 0.05, ^∗∗^*p* < 0.01, NS no significance. LG = LPS+galactosamine. MLG = midazolam+LPS+galactosamine. Mida = midazolam.

The mice serum ALT levels were elevated in the LG group but were reduced in the MLG group (Figure 1A). Histological analyses of mouse liver showed that in the Control group and the Mida group no inflammation and necrosis were found. LPS plus galactosamine treatment (LG group) resulted in spotty coagulation necrosis within the lobules preferentially located in the perivenular areas, and a mild inflammatory cells infiltration within the necrosis area, consisting of neutrophils and mononuclear cells. However, midazolam treated mice (MLG group) exhibited no necrosis and inflammation in the liver tissue although the mice received same dose of LPS and galactosamine (Figure 1B). These results revealed that midazolam prevented the liver injury caused by LPS plus galactosamine and protected liver function to some extent as well.

In real-time PCR detection of liver tissues, the relative mRNA levels of CCR2, TNF-α, and IL-1β were increased in LG mice but decreased dramatically in MLG mice (Figure 1C). Midazolam intensified Arg-1 transcription in the livers of MLG group, whereas there was no difference in Arg-1 transcription between the LG group and the Control group (Figure 1C). Midazolam also remarkably reduced iNOSmRNA expression in the livers of MLG mice, although the augmentation of iNOSmRNA in the LG group was not significantly different from that in the Control group (Figure 1C). These PCR data suggested that midazolam might limit the liver inflammation by suppressing pro-inflammatory cytokines, such as TNF-α, IL-1β, and iNOS, and might promote anti-inflammatory reactions by boosting Arg-1 expression.

The ALT levels and mRNA expressions had no apparent differences between the Control group and the Mida group (Figure 1A,C).

### Midazolam Inhibited Liver Inflammatory Responses Mainly by Its Effects on Liver Macrophages

**FIGURE 2:**
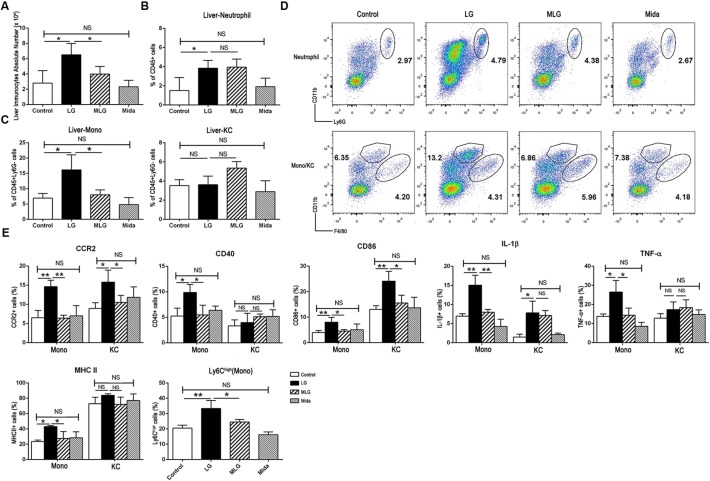
Midazolam reduced liver infiltrating monocytes and inhibited the production of the inflammatory molecules by liver macrophages especially by liver infiltrating monocytes. **(A)** The number of liver immunocytes in each group. Statistical analysis of the proportion of **(B)** liver neutrophils, and **(C)** liver infiltrating monocytes (Liver-Mono) and Kupffer cells (Liver-KC), measured by flow cytometry. **(D)** Representative flow cytometry images of liver neutrophils (upper), infiltrating monocytes and Kupffer cells (lower) in the liver tissues of each group. **(E)** Statistical analysis of inflammatory molecules produced by liver infiltrating monocytes (Mono) and Kupffer cells (KC), respectively, which were measured by flow cytometry. The data are presented as the mean ± *SD*, *n* = 4 in each group. The difference among groups was examined by one-way ANOVA analysis and comparison between two groups was analyzed by *t*-test. ^∗^*p* < 0.05, ^∗∗^*p* < 0.01, NS no significance. LG = LPS+galactosamine. MLG = midazolam+LPS+galactosamine. Mida = midazolam.

In LPS plus galactosamine induced liver injury, the number of liver immunocytes, the proportions of liver infiltrating monocytes (Liver-Mono, CD45^+^Ly6G^-^CD11b^high^F4/80^low^) and neutrophils (Liver-Neu, CD45^+^CD11b^+^Ly6G^+^) were increased markedly; and midazolam led to a significant reduction in the number of liver immunocytes and the proportion of infiltrating monocytes, but did not affect intrahepatic neutrophils. Also, the proportion of Kupffer cells (CD45^+^Ly6G^-^CD11b^low^F4/80^high^) was not affect by LPS and galactosamine; and midazolam expanded this portion but without significance (Figure 2A–D). Based on the evidences above, we speculated that midazolam might restrict liver innate inflammatory responses mainly by its effect on liver macrophages.

We examined pro-inflammatory molecules and cell markers in Kupffer cells and liver infiltrating monocytes by flow cytometry. Compared with the control group, LPS plus galactosamine obviously upregulated the expression of CCR2, IL-1β, and CD86 in both types of cells, while MHC II, CD40, and TNF-α were only increased in the infiltrating monocytes; the elevations were reversed by midazolam except the IL-1β in Kupffer cells (Figure 2E). We further explored the fraction of infiltrating Ly6C^high^ monocytes; midazolam led to a reduction in such populations in LPS plus galactosamine injured livers (Figure 2E). The results indicated that midazolam might have a greater impact on liver infiltrating monocytes than on Kupffer cells.

### Midazolam Inhibited Pro-inflammatory Response in BM Monocytes Cultured *in vitro*

**FIGURE 3:**
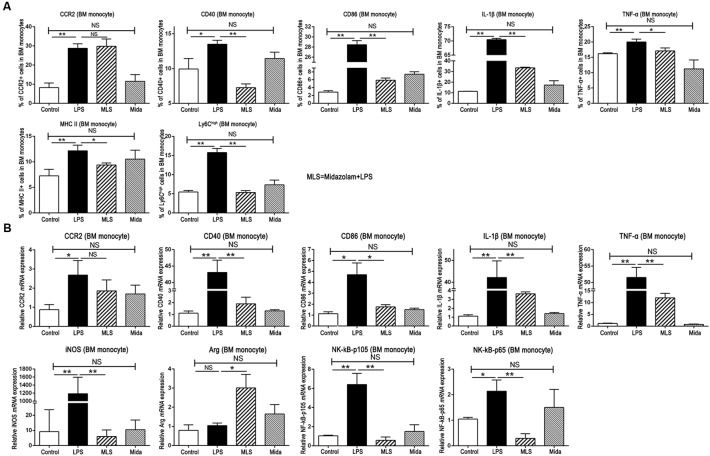
Effect of midazolam on BM cells cultured *in vitro*. **(A)** Statistical analysis of inflammatory molecules produced by the cultured BM cells, which were measured by flow cytometry. **(B)** Relative mRNA expression levels in the cultured BM cells, which were detected by real-time PCR. The data are presented as the mean ± *SD*, *n* = 3 in each group. The difference among groups was examined by one-way ANOVA analysis and comparison between two groups was analyzed by *t*-test. ^∗^*p* < 0.05, ^∗∗^*p* < 0.01, NS no significance. LPS = lipopolysaccharide. MLS = midazolam+LPS. Mida = midazolam.

Due to the origin of liver infiltrating monocytes, we extracted the BM cells from the naïve C57BL/6 mice and cultured them in complete medium with or without midazolam followed by LPS stimulation. The BM monocytes were analyzed by flow cytometry. Compared with the Control group, LPS increased the products of TNF-α, IL-1β, CD40, CD86, CCR2, and MHC II by BM monocytes, and promoted the proportion of Ly6C^high^ monocytes. Midazolam substantially suppressed the high expression of those molecules except CCR2 and inhibited the enhancement of Ly6C^high^ monocytes (Figure 3A).

The real-time PCR results from the cultured BM cells had a tendency similar to FACS. In detail, LPS remarkably upregulated the mRNA transcripts of TNF-α, IL-1β, CD40, CD86, and CCR2, whereas midazolam downregulated those gene expressions with significance except CCR2 (Figure 3B). Moreover, midazolam upregulated the Arg-1mRNA in LPS stimulated BM cells, while LPS alone had no such effect. Contrary to Arg-1, midazolam markedly inhibited iNOS transcription in LPS stimulated BM cells (Figure 3B). We noticed that these changes of Arg-1 and iNOS acquired *in vitro* were similar to those obtained *in vivo*, verifying that midazolam could restrain the pro-inflammatory function of macrophages in response to LPS. In addition, gene transcripts of NF-κB subunits p105 and p65, which were upregulated after LPS stimulation, were remarkably downregulated in the presence of midazolam (Figure 3B). The mRNA alteration of NF-κB subunits was parallel to that of TNF-α and IL-1β described in this study, suggesting that the inhibitory effect of midazolam on inflammatory responses of macrophage might partially be due to the attenuation of NF-κB activity by the reduction of p105 and p65 mRNA transcription.

### The Anti-inflammatory Effect of Midazolam on BM Monocytes Was Exerted via the PBR

**FIGURE 4:**
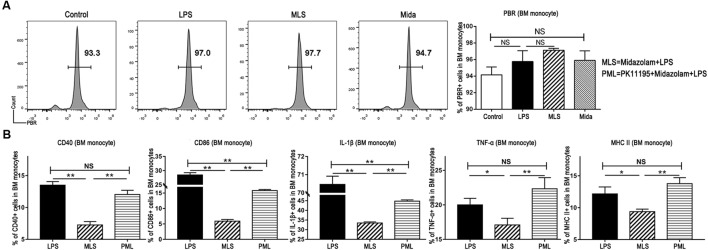
Midazolam might exert its anti-inflammatory function via PBR in the BM monocytes. **(A)** Representative flow cytometry images and statistical analysis of PBR in the cultured BM monocytes. **(B)** Statistical analysis of inflammatory molecules produced by BM monocytes pretreated with or without PK-11195, which were measured by flow cytometry. The data are presented as the mean ± *SD*, *n* = 3 in each group. The difference among groups was examined by one-way ANOVA analysis and comparison between two groups was analyzed by *t*-test. ^∗^*p* < 0.05, ^∗∗^*p* < 0.01, NS no significance. LPS = lipopolysaccharide. MLS = midazolam+LPS. PML = PK11195+midazolam+LPS.

It is well known that the binding sites of midazolam exist inside the central nerve system and also in peripheral tissues (Kaynar et al., 2013). Our flow cytometry results exhibited the existence of the PBR in BM monocytes (Figure 4A). Therefore, we studied the cultured BM monocytes in the presence and the absence of PK-11195. PK-11195, a specific PBR blocker, almost fully switched TNF-α, CD40, and MHC II to the previous levels induced by LPS, and promoted IL-1β and CD86 to a level obviously higher than that in the absence of PK-11195 (Figure 4B). These results demonstrated that midazolam could inhibit the inflammatory response by binding to the PBR in the macrophages.

## Discussion

In the inflamed liver, the activated immunity and the excessive inflammation are always accompanied by liver tissue injury and liver dysfunction (Possamai et al., 2014; Heymann and Tacke, 2016). In this study, LPS plus galactosamine treated mice had an apparent elevation of serum ALT and classical histopathological manifestations of liver injury. Consistent with the acute liver injury, there was an accumulation of inflammatory cells and increased gene expression of pro-inflammatory cytokines. Compared to the mice treated by LPS plus galactosamine, the serum ALT was decreased, and liver histological features had no apparent damage in the mice pre-treated with midazolam followed by LPS and galactosamine. It suggested that midazolam might have preventive effect on LPS and galactosamine induced liver injure. We also found that midazolam decreased TNF-α and IL-1β mRNA transcripts and reduced the fraction of liver infiltrating monocytes. These findings suggested that midazolam might protect the liver by suppressing the immuno-inflammatory responses in the liver. However, midazolam did not display an inhibition on liver neutrophils. This finding led us to speculate that midazolam might protect liver mainly by its effects on liver macrophages.

According to the difference of origin, liver macrophages are composed of resident Kupffer cells and infiltrating monocytes from circulation (Karlmark et al., 2009; Tacke and Zimmermann, 2014; Ju and Tacke, 2016). In the normal liver, Kupffer cells prevail in the macrophage pool and preserve immunological tolerance (Karlmark et al., 2009). In the injured liver, bone marrow-derived monocytes infiltrate into the liver and differentiate into macrophages, then dominate the subsequent inflammation together with the Kupffer cells (Dal-Secco et al., 2015). In the current study, liver macrophages were activated in the LPS plus galactosamine treated mice with an accumulation of TNF-α and IL-1β; and midazolam hindered macrophages from escalating inflammatory responses by suppressing the production of these two cytokines in liver infiltrating monocytes. The findings suggested that midazolam had the ability to downregulate the innate immunity and pro-inflammatory action of the macrophages in response to inflammatory insults.

MHC II (Antoniades et al., 2008; Varin and Gordon, 2009), together with costimulatory molecules CD86 (Yang et al., 2013; Ruiz-Rosado Jde et al., 2016) and CD40 (Seino and Taniguchi, 2005; Shiba et al., 2016) on the macrophages are essential in antigen presentation, which can activate T cells and elicit subsequent cellular and humoral immune responses during the liver injury (Kimura et al., 2006; Antoniades et al., 2008; Elgueta et al., 2009). In our study, the expressions of MHC II, CD40, and CD86 on the liver macrophages were elevated in LPS and galactosamine injured liver, and depressed in the treatment of midazolam; and the change of these molecules was mainly found in the liver infiltrating monocytes. These results suggested that midazolam impeded the inherent ability of antigen presentation in liver macrophages.

Numerous murine liver injury models indicate that the migration and accumulation of macrophages in the injured liver are dependent on CCR2 (Zigmond et al., 2014; Mossanen et al., 2016; Wang et al., 2016). Our study showed that CCR2mRNA was increased in the injured liver, specifically, CCR2 was highly expressed on both Kupffer cells and liver infiltrating monocytes in the LPS plus galactosamine treated mice. With the treatment of midazolam, CCR2 was reduced both in the injured liver and on liver macrophages, which might impede chemotaxis and migration of macrophages.

Ly6C^high^ monocytes are often massively recruited into the injured area of liver, and are considered to be pro-inflammatory M1 phenotype (Karlmark et al., 2009; Zigmond et al., 2012). With the treatment of midazolam, the fraction of Ly6C^high^ monocytes in the mouse liver was reduced.

Our data from the *in vivo* experiments revealed that midazolam could prevent liver injury probably via regulating liver macrophage functions, such as pro-inflammatory cytokines production, antigen presentation, and macrophage migration. Further, we found that midazolam had a greater impact on liver infiltrating monocytes than on Kupffer cells.

To verify the results acquired *in vivo*, we further isolated BM cells and cultured in the presence or absence of midazolam followed by LPS stimulation. For the BM monocytes, midazolam had similar effects *in vitro* as to those *in vivo*, except for CCR2.

Increasing evidence demonstrates that liver macrophages have pro-inflammatory actions (M1) in pathogen elimination and tissue destruction, and also possess anti-inflammatory properties (M2) in resolving inflammation and promoting tissue repair (Ramachandran et al., 2012; Sica et al., 2014). Since the M1 macrophages advocate the Th1 immune response in association with iNOS (Karlmark et al., 2009; Raber et al., 2012) and M2 can reduce inflammation by upregulating Arg-1 (Cassetta et al., 2011), we detected the mRNA of iNOS and Arg-1 in the mice livers and cultured BM cells. In the LPS plus galactosamine injured livers, the mRNA level of iNOS was increased, whereas the Arg-1 remained steady; when the liver injured mice were treated with midazolam, the mRNA of iNOS was decreased, but Arg-1 was upregulated. Similar changes of iNOS and Arg-1 were also seen in the cultured BM cells. These results suggested that midazolam could facilitate monocytes to switch the phenotype into M2 with an imaginable reduction of inflammation in the liver.

NF-κB is a major intracellular signaling pathway involved in the progress of inflammation. The activated NF-κB pathway may result in the products of pro-inflammatory cytokines and mediators (O’Neill and Bowie, 2007; Qin et al., 2016). In our study, we found a massive production of pro-inflammatory cytokines (such as TNF-α, IL-1β, and iNOS) and increased antigen presenting molecules (such as MHC II, CD86, and CD40) both in the liver macrophages stimulated by LPS and galactosamine *in vivo* and in the BM monocytes agitated by LPS *in vitro*. Consistent with the changes above, the mRNA levels of NF-κB components p105 and p65 were also increased in the LPS stimulated BM cells, indicating the activation of NF-κB. These findings are consistent with the results in different experiments (Krappmann et al., 2004; Hansen et al., 2015; Qin et al., 2016; Park et al., 2017). With the treatment of midazolam, the escalated inflammation was suppressed both *in vivo* and *in vitro*, and the hindered pro-inflammatory responses of monocytes *in vitro* were accompanied by a decrease in mRNA transcripts of NF-κB. Kim et al. found that midazolam inhibited NF-κB activation in LPS-stimulated RAW264.7 cells by blocking IκBα degradation and inhibiting translocation of NF-κB p65 subunit into the nucleus (Kim et al., 2006). Therefore, we speculated that the anti-inflammatory effects of midazolam on macrophages were exerted, at least in part, by blocking the NF-κB pathway.

Midazolam has the equal ability in binding to the PBR and to the receptors in central nerve system. Therefore, we first detected the existence of PBR in the BM monocytes by flow cytometry. With a positive outcome, we subsequently applied PK-11195, an antagonist of PBR, to the cultured monocytes. Then we found that TNF-α, IL-1β, MHC II, CD86, CD40 and the fraction of Ly6C^high^ monocytes, which were downregulated by midazolam in the LPS-stimulated monocytes, were upregulated by PK-11195. These reversal manifestations of PK-11195 indicated that binding to the PBR was responsible for the anti-inflammatory effect of midazolam on LPS-activated macrophages.

In summary, midazolam can prevent LPS induced immune mediated liver injury by suppressing the immune response of liver macrophages, and polarizing monocytes/macrophages from pro-inflammatory M1 to anti-inflammatory M2. These results can be, at least partially, attributed to the reduced activity in the NF-κB pathway after midazolam binding to the PBR in the macrophages.

## Author Contributions

All listed authors participated meaningfully in this study and that they have seen and approved the submission of this manuscript. JL, HT, and XZ participated in performing the research, analyzing the data, and initiating the original draft of the article. CZ, HJ, YT, XZ, and XL participated in performing the research and collecting the data. DZ, MD, and XS established the hypotheses, supervised the studies, analyzed the data, and co-wrote the manuscript.

## Conflict of Interest Statement

The authors declare that the research was conducted in the absence of any commercial or financial relationships that could be construed as a potential conflict of interest.

## Abbreviations

ALT: alanine transaminase
Arg-1: Arginase-1
BM: bone marrow
CCR2: CC chemokine receptor 2
CD40: cluster of differentiation 40
CD86: cluster of differentiation 86
DMSO: dimethyl sulfoxide
FACS: Fluorescence-activated cell sorter
GAPDH: glyceraldehyde-3-phosphate dehydrogenase.**;** IL-1β interleukin-1β
iNOS: inducible nitric oxide synthase
LPS: lipopolysaccharide
MHC II: major histocompatibility complex class II
NF-κB: nuclear factor-κB
NS: normal saline
PBR: peripheral benzodiazepine receptor
PCR: polymerase chain reaction
Th1: T helper 1
TNF-α: tumor necrosis factor alpha

## References

[B1] Anderson L. E. Dring A. M. Hamel L. D. Stoner M. A. (2011). Modulation of constitutive androstane receptor (CAR) and pregnane X receptor (PXR) by 6-arylpyrrolo[2,1-d][1,5]benzothiazepine derivatives, ligands of peripheral benzodiazepine receptor (PBR). *Toxicol. Lett.* 202 148–154. 10.1016/j.toxlet.2011.02.004 21315811 PMC3086002

[B2] Antoniades C. G. Berry P. A. Wendon J. A. Vergani D. (2008). The importance of immune dysfunction in determining outcome in acute liver failure. *J. Hepatol.* 49 845–861. 10.1016/j.jhep.2008.08.009 18801592

[B3] Cassetta L. Cassol E. Poli G. (2011). Macrophage polarization in health and disease. *ScientificWorldJournal* 11 2391–2402. 10.1100/2011/213962 22194670 PMC3236674

[B4] Cheng B. Xie G. Yao S. Wu X. Guo Q. Gu M. (2007). Epidemiology of severe sepsis in critically ill surgical patients in ten university hospitals in China. *Crit. Care Med.* 35 2538–2546. 10.1097/01.CCM.0000284492.30800.00 17828034

[B5] Dal-Secco D. Wang J. Zeng Z. Kolaczkowska E. Wong C. H. Petri B. (2015). A dynamic spectrum of monocytes arising from the in situ reprogramming of CCR2+ monocytes at a site of sterile injury. *J. Exp. Med.* 212 447–456. 10.1084/jem.20141539 25800956 PMC4387291

[B6] Elgueta R. Benson M. J. de Vries V. C. Wasiuk A. Guo Y. Noelle R. J. (2009). Molecular mechanism and function of CD40/CD40L engagement in the immune system. *Immunol. Rev.* 229 152–172. 10.1111/j.1600-065X.2009.00782.x 19426221 PMC3826168

[B7] Hansen F. C. Kalle-Brune M. van der Plas M. J. Stromdahl A. C. Malmsten M. Morgelin M. (2015). The thrombin-derived host defense peptide gky25 inhibits endotoxin-induced responses through interactions with Lipopolysaccharide and Macrophages/Monocytes. *J. Immunol.* 194 5397–5406. 10.4049/jimmunol.1403009 25911750

[B8] Helmy S. A. Al-Attiyah R. J. (2001). The immunomodulatory effects of prolonged intravenous infusion of propofol versus midazolam in critically ill surgical patients. *Anaesthesia* 56 4–8. 11167428 10.1046/j.1365-2044.2001.01713.x

[B9] Heymann F. Tacke F. (2016). Immunology in the liver–from homeostasis to disease. *Nat. Rev. Gastroenterol. Hepatol.* 13 88–110. 10.1038/nrgastro.2015.200 26758786

[B10] Ju C. Tacke F. (2016). Hepatic macrophages in homeostasis and liver diseases: from pathogenesis to novel therapeutic strategies. *Cell Mol. Immunol.* 13 316–327. 10.1038/cmi.2015.104 26908374 PMC4856798

[B11] Karlmark K. R. Weiskirchen R. Zimmermann H. W. Gassler N. Ginhoux F. Weber C. (2009). Hepatic recruitment of the inflammatory Gr1+ monocyte subset upon liver injury promotes hepatic fibrosis. *Hepatology* 50 261–274. 10.1002/hep.22950 19554540

[B12] Kaynar G. Yurdakan G. Comert F. Yilmaz-Sipahi E. (2013). Effects of peripheral benzodiazepine receptor ligand Ro5-4864 in four animal models of acute lung injury. *J. Surg. Res.* 182 277–284. 10.1016/j.jss.2012.10.023 23127280

[B13] Kim S. N. Son S. C. Lee S. M. Kim C. S. Yoo D. G. Lee S. K. (2006). Midazolam inhibits proinflammatory mediators in the lipopolysaccharide-activated macrophage. *Anesthesiology* 105 105–110. 16810001 10.1097/00000542-200607000-00019

[B14] Kimura K. Moriwaki H. Nagaki M. Saio M. Nakamoto Y. Naito M. (2006). Pathogenic role of B cells in anti-CD40-induced necroinflammatory liver disease. *Am. J. Pathol.* 168 786–795. 10.2353/ajpath.2006.050314 16507894 PMC1606511

[B15] Kobashi H. Toshimori J. Yamamoto K. (2013). Sepsis-associated liver injury: Incidence, classification and the clinical significance. *Hepatol. Res.* 43 255–266. 10.1111/j.1872-034X.2012.01069.x 22971102

[B16] Krappmann D. Wegener E. Sunami Y. Esen M. Thiel A. Mordmuller B. (2004). The IkappaB kinase complex and NF-kappaB act as master regulators of lipopolysaccharide-induced gene expression and control subordinate activation of AP-1. *Mol. Cell. Biol.* 24 6488–6500. 10.1128/MCB.24.14.6488-6500.2004 15226448 PMC434242

[B17] Mossanen J. C. Krenkel O. Ergen C. Govaere O. Liepelt A. Puengel T. (2016). Chemokine (C-C motif) receptor 2-positive monocytes aggravate the early phase of acetaminophen-induced acute liver injury. *Hepatology* 64 1667–1682. 10.1002/hep.28682 27302828

[B18] Ohta N. Ohashi Y. Takayama C. Mashimo T. Fujino Y. (2011). Midazolam suppresses maturation of murine dendritic cells and priming of lipopolysaccharide-induced t helper 1-type immune response. *Anesthesiology* 114 355–362. 10.1097/ALN.0b013e3182070c1f 21245731

[B19] O’Neill L. A. Bowie A. G. (2007). The family of five: TIR-domain-containing adaptors in Toll-like receptor signalling. *Nat. Rev. Immunol.* 7 353–364. 10.1038/nri2079 17457343

[B20] Park H. S. Nelson D. E. Taylor Z. E. Hayes J. B. Cunningham K. D. Arivett B. A. (2017). Suppression of LPS-induced NF-kappaB activity in macrophages by the synthetic aurone, (Z)-2-((5-(hydroxymethyl) furan-2-yl) methylene) benzofuran-3(2H)-one. *Int. Immunopharmacol.* 43 116–128. 10.1016/j.intimp.2016.12.004 27988459

[B21] Possamai L. A. Thursz M. R. Wendon J. A. Antoniades C. G. (2014). Modulation of monocyte/macrophage function: a therapeutic strategy in the treatment of acute liver failure. *J. Hepatol.* 61 439–445. 10.1016/j.jhep.2014.03.031 24703954

[B22] Qin X. Jiang X. Jiang X. Wang Y. Miao Z. He W. (2016). Micheliolide inhibits LPS-induced inflammatory response and protects mice from LPS challenge. *Sci. Rep.* 6:23240. 10.1038/srep23240 26984741 PMC4794649

[B23] Raber P. Ochoa A. C. Rodriguez P. C. (2012). Metabolism of L-arginine by myeloid-derived suppressor cells in cancer: mechanisms of T cell suppression and therapeutic perspectives. *Immunol. Invest.* 41 614–634. 10.3109/08820139.2012.680634 23017138 PMC3519282

[B24] Ramachandran P. Pellicoro A. Vernon M. A. Boulter L. Aucott R. L. Ali A. (2012). Differential Ly-6C expression identifies the recruited macrophage phenotype, which orchestrates the regression of murine liver fibrosis. *Proc. Natl. Acad. Sci. U.S.A.* 109 E3186–E3195. 10.1073/pnas.1119964109 23100531 PMC3503234

[B25] Ruiz-Rosado Jde D. Olguin J. E. Juarez-Avelar I. Saavedra R. Terrazas L. I. Robledo-Avila F. H. (2016). MIF Promotes Classical Activation and Conversion of Inflammatory Ly6C(high) Monocytes into TipDCs during Murine Toxoplasmosis. *Mediators Inflamm.* 2016:9101762. 10.1155/2016/9101762 27057101 PMC4789477

[B26] Seino K. Taniguchi M. (2005). Functionally distinct NKT cell subsets and subtypes. *J. Exp. Med.* 202 1623–1626. 10.1084/jem.20051600 16365145 PMC2212962

[B27] Shiba T. Makino I. Kawakami K. Kato I. Kobayashi T. Kaneko K. (2016). p-Cresyl sulfate suppresses lipopolysaccharide-induced anti-bacterial immune responses in murine macrophages in vitro. *Toxicol. Lett.* 245 24–30. 10.1016/j.toxlet.2016.01.009 26784855

[B28] Sica A. Invernizzi P. Mantovani A. (2014). Macrophage plasticity and polarization in liver homeostasis and pathology. *Hepatology* 59 2034–2042. 10.1002/hep.26754 24115204

[B29] Tacke F. Zimmermann H. W. (2014). Macrophage heterogeneity in liver injury and fibrosis. *J. Hepatol.* 60 1090–1096. 10.1016/j.jhep.2013.12.025 24412603

[B30] van Gestel A. Bakker J. Veraart C. P. van Hout B. A. (2004). Prevalence and incidence of severe sepsis in Dutch intensive care units. *Crit. Care* 8 R153–R162. 10.1186/cc2858 15312213 PMC522831

[B31] Varin A. Gordon S. (2009). Alternative activation of macrophages: immune function and cellular biology. *Immunobiology* 214 630–641. 10.1016/j.imbio.2008.11.009 19264378

[B32] Wang T. Wang Z. Yang P. Xia L. Zhou M. Wang S. (2016). PER1 prevents excessive innate immune response during endotoxin-induced liver injury through regulation of macrophage recruitment in mice. *Cell Death Dis.* 7:e2176. 10.1038/cddis.2016.9 27054331 PMC4855679

[B33] Yan J. Li S. Li S. (2014). The role of the liver in sepsis. *Int. Rev. Immunol.* 33 498–510. 10.3109/08830185.2014.889129 24611785 PMC4160418

[B34] Yang Y. Zhang R. Xia F. Zou T. Huang A. Xiong S. (2013). LPS converts Gr-1(+)CD115(+) myeloid-derived suppressor cells from M2 to M1 via P38 MAPK. *Exp. Cell Res.* 319 1774–1783. 10.1016/j.yexcr.2013.05.007 23701951

[B35] Zhang J. Dong Z. Zhou R. Luo D. Wei H. Tian Z. (2005). Isolation of lymphocytes and their innate immune characterizations from liver, intestine, lung and uterus. *Cell Mol. Immunol.* 2 271–280. 16274625

[B36] Zigmond E. Samia-Grinberg S. Pasmanik-Chor M. Brazowski E. Shibolet O. Halpern Z. (2014). Infiltrating monocyte-derived macrophages and resident kupffer cells display different ontogeny and functions in acute liver injury. *J. Immunol.* 193 344–353. 10.4049/jimmunol.1400574 24890723

[B37] Zigmond E. Varol C. Farache J. Elmaliah E. Satpathy A. T. Friedlander G. (2012). Ly6C hi monocytes in the inflamed colon give rise to proinflammatory effector cells and migratory antigen-presenting cells. *Immunity* 37 1076–1090. 10.1016/j.immuni.2012.08.026 23219392

