# Supplementary material for: The Protection of Midazolam Against Immune Mediated Liver Injury Induced by Lipopolysaccharide and Galactosamine in Mice
Source: Front Pharmacol. 2019 Jan 8;9:1528. doi: 10.3389/fphar.2018.01528 (PMC6331471; doi:10.3389/fphar.2018.01528)
Supplement: Supplementary file 1 [file Data_Sheet_1.docx]

Supplementary Material

**The protection of midazolam against immune mediated liver injury induced by lipopolysaccharide and galactosamine in mice**

**Running title: Midazolam protects immune mediated liver injury**

Jian Li, Hong Tan, Xiaona Zhou, Chunpan Zhang, Hua Jin, Yue Tian, Xinyan Zhao, Xinmin Li, Xuelian Sun^*^, Meili Duan^*^ and Dong Zhang^*^

***Corresponding Authors:**

Dong Zhang: [zhangd2010@hotmail.com](mailto:zhangd2010@hotmail.com)

Meili Duan: [beauty9659@hotmail.com](mailto:beauty9659@hotmail.com)

Xuelian Sun: [sunxuelian11@163.com](mailto:sunxuelian11@163.com)

**Lead Contact:** Dong Zhang, [zhangd2010@hotmail.com](mailto:zhangd2010@hotmail.com)

# Supplementary Figures and Tables

## Supplementary Figure S1


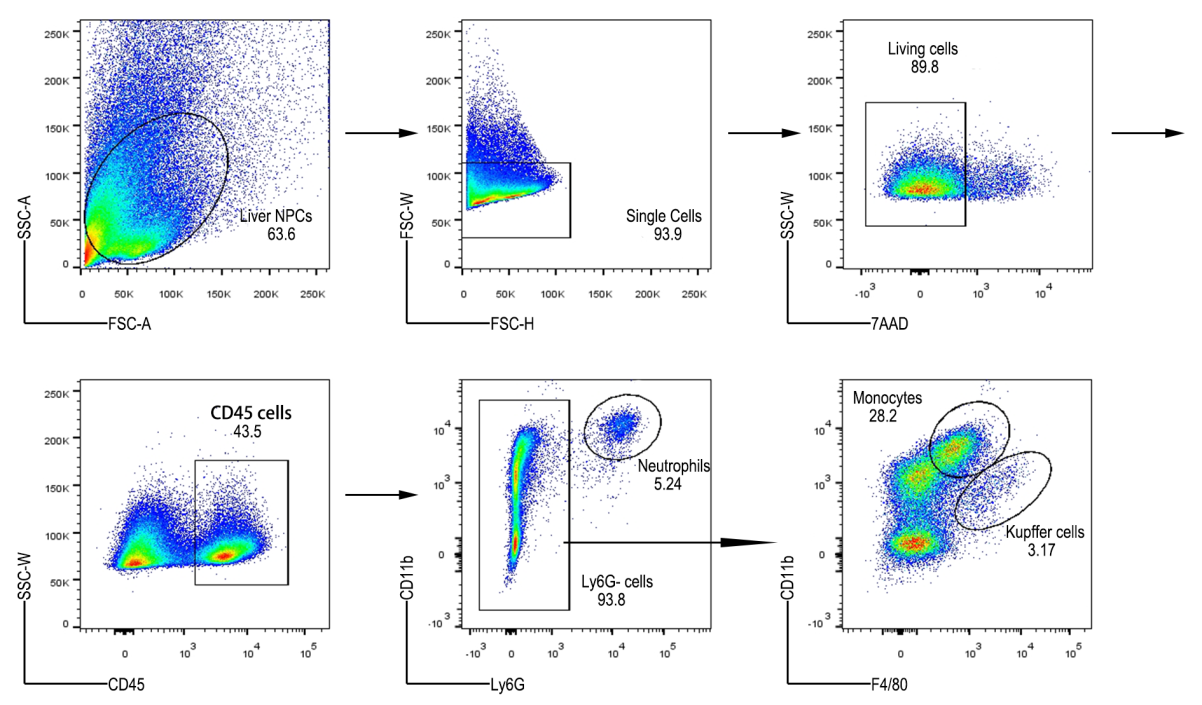


## Supplementary Figure S1. Representative flow cytometry images of the gating strategy used for flow cytometry analysis on liver immunocytes. Liver NPCs, liver nonparenchymal cells.

## Supplementary Figure S2


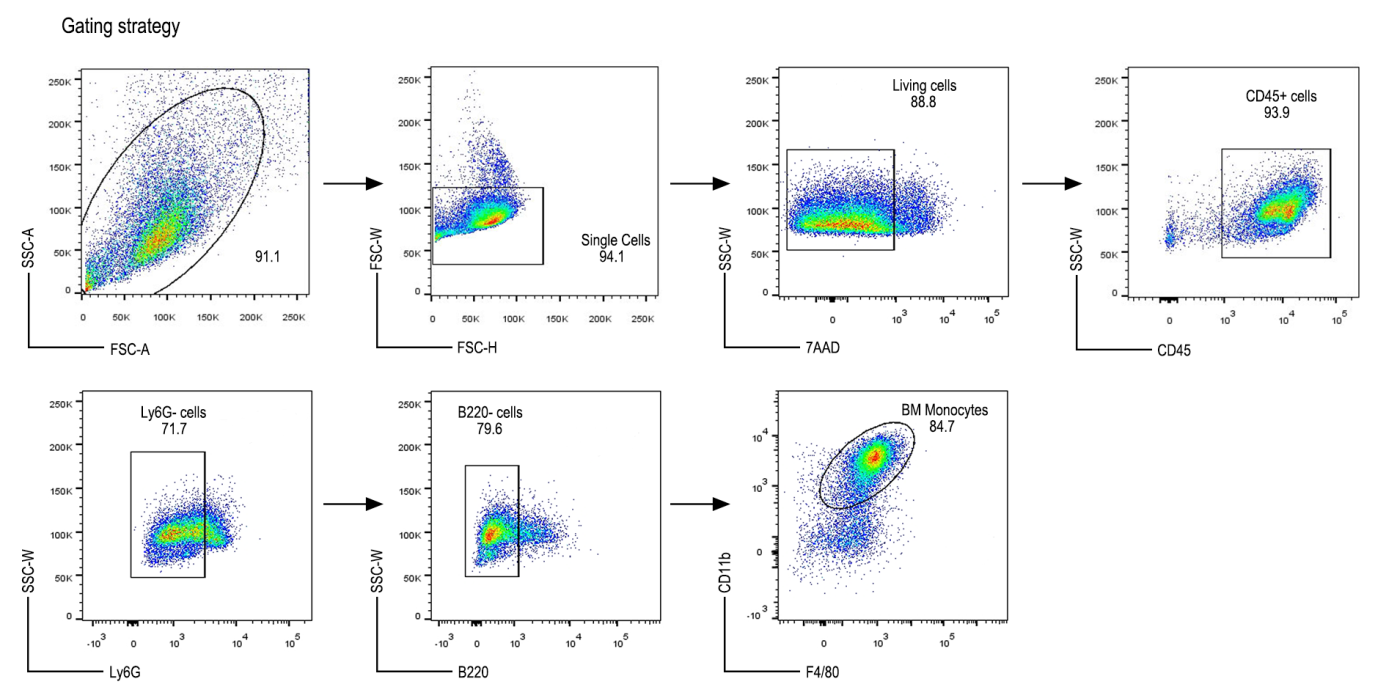


## Supplementary Figure S2. Representative flow cytometry images of the gating strategy used for flow cytometry analysis on BM monocytes. BM, bone marrow.

**Supplementary Table S1 Antibodies and Experimental Information**

| **Antibodies** | **Clone** | **Source** | **Identifier** | **Dilution** | **Blocking conditions** |
| --- | --- | --- | --- | --- | --- |
| PE anti-TER119 | TER-119 | Thermo Fisher Scientific | Cat#: 12-5921-82 | 1/100 | Mouse FcR Blocker, 30min/4 ºC |
| PE anti-GR1 | RB6-8C5 | Thermo Fisher Scientific | Cat#: 12-5931-83 | 1/100 | Mouse FcR Blocker, 30min/4 ºC |
| PE anti-B220 | RA3-6B2 | Thermo Fisher Scientific | Cat#: 12-0452-82 | 1/100 | Mouse FcR Blocker, 30min/4 ºC |
| PE/Cy7  anti-CD11b | M1/70 | Thermo Fisher Scientific | Cat# 25-0112-82 | 1/200 | Mouse FcR Blocker, 30min/4 ºC |
| FITC anti-CD11b | M1/70 | Thermo Fisher Scientific | Cat# 11-0112-82 | 1/200 | Mouse FcR Blocker, 30min/4 ºC |
| PE anti-CD11b | M1/70 | Thermo Fisher Scientific | Cat# 12-0112-85 | 1/200 | Mouse FcR Blocker, 30min/4 ºC |
| PE/Cy7  anti-CD45 | 30-F11 | Thermo Fisher Scientific | Cat#: 25-0451-82 | 1/200 | Mouse FcR Blocker, 30min/4 ºC |
| PE anti-CD45 | 30-F11 | Thermo Fisher Scientific | Cat#: 12-0451-82 | 1/200 | Mouse FcR Blocker, 30min/4 ºC |
| PercP-Cy5.5  anti-Ly6G | 1A8 | Biolegend | Cat#: 127616 | 1/200 | Mouse FcR Blocker, 30min/4 ºC |
| APC/Cy7  anti-Ly6G | 1A8 | Biolegend | Cat#: 127624 | 1/200 | Mouse FcR Blocker, 30min/4 ºC |
| APC anti-F4/80 | BM8 | Thermo Fisher Scientific | Cat#: 17-4801-82 | 1/200 | Mouse FcR Blocker, 30min/4 ºC |
| PercP-Cy5.5  anti-F4/80 | BM8 | Thermo Fisher Scientific | Cat#: 45-4801-82 | 1/200 | Mouse FcR Blocker, 30min/4 ºC |
| APC anti-CCR2 | SA203G11 | Biolegend | Cat#: 150603 | 1/200 | Mouse FcR Blocker, 30min/4 ºC |
| FITC anti-CCR2 | SA203G11 | Biolegend | Cat#: 150607 | 1/200 | Mouse FcR Blocker, 30min/4 ºC |
| PE/Cy7  anti-CD86 | GL1 | Thermo Fisher Scientific | Cat#: 25-0862-82 | 1/200 | Mouse FcR Blocker, 30min/4 ºC |
| APC anti-CD40 | HM40-3 | Thermo Fisher Scientific | Cat#: 17-0402-82 | 1/200 | Mouse FcR Blocker, 30min/4 ºC |
| APC anti-MHC II | M5/114.15.2 | Thermo Fisher Scientific | Cat#: 17-5321-82 | 1/200 | Mouse FcR Blocker, 30min/4 ºC |
| PE anti-TNFα | MP6-XT22 | Thermo Fisher Scientific | Cat#: 12-7321-82 | 1/200 | Mouse FcR Blocker, 30min/4 ºC |
| APC anti-Ly6C | HK1.4 | Thermo Fisher Scientific | Cat#: 17-5932-82 | 1/200 | Mouse FcR Blocker, 30min/4 ºC |
| Biotin anti-IL-1β | B122 | Biolegend | Cat#: 503505 | 1/100 | Mouse FcR Blocker, 30min/4 ºC |
| APC Streptavidin | / | Biolegend | Cat#: 405207 | 1/100 | / |
| Rabbit IgM Anti-mouse PBR | EPR5384 | Abcam | ab109497 | 1/50 | 1x PBS / 10% normal goat serum / 0.3M glycine, 30min/RT |
| Alexa Fluor®647 Goat pAb to Rb IgM | / | Abcam | ab150095 | 1/2000 | / |

## Supplementary Table S2. Primer Sequences Used In Real-Time PCR

| Gene |  | Strand |  | Primer sequence (5'-3') |
| --- | --- | --- | --- | --- |
| *TNF-a* |  | Sense |  | TCCCAGGTTCTCTTCAAGGGA |
|  |  | Antisense |  | GGTGAGGAGCACGTAGTCGG |
| *IL-1β* |  | Sense |  | CCCTGCAGCTGGAGAGTGTGGA |
|  |  | Antisense |  | TGTGCTCTGCTTGTGAGGTGCTG |
| *CCR2* |  | Sense |  | TTTGTTTTTGCAGATGATTCAA |
|  |  | Antisense |  | TGCCATCATAAAGGAGCCAT |
| *CD40* |  | Sense |  | GTTTAAAGTCCCGGATGCGA |
|  |  | Antisense |  | CTCAAGGCTATGCTGTCTGT |
| *CD86* |  | Sense |  | ACGATGGACCCCAGATGCACCA |
|  |  | Antisense |  | GCGTCTCCACGGAAACAGCA |
| *Arg-1* |  | Sense |  | TGACTGAAGTAGACAAGCTGGGGAT |
|  |  | Antisense |  | CGACATCAAAGCTCAGGTGAATCGG |
| *iNOS* |  | Sense |  | ATCTTTGCCACCAAGATGGCCTGG |
|  |  | Antisense |  | TTCCTGTGCTGTGCTACAGTTCCG |
| *NF-κB1 (p105)* |  | Sense |  | GTGACAGTGGTGTGGAGACATC |
|  |  | Antisense |  | GGGGCATTTTGTTCAGAGATAG |
| *p65 (Rel A)* |  | Sense |  | GGATGGCTACTATGAGGCTGAC |
|  |  | Antisense |  | AGGTCTCGCTTCTTCACACACT |
| *GAPDH* |  | Sense |  | AAGGTCATCCCAGAGCTGAA |
|  |  | Antisense |  | CTGCTTCACCACCTTCTTGA |
